# What are the factors associated with people with advanced dementia refusing assistance with personal care?

**DOI:** 10.1002/gps.5857

**Published:** 2022-12-09

**Authors:** Tamara Backhouse, Anne Killett, Eneida Mioshi, Mizanur Khondoker

**Affiliations:** ^1^ School of Health Sciences University of East Anglia Norwich Norfolk UK; ^2^ Norwich Medical School University of East Anglia Norwich Norfolk UK

**Keywords:** care home, dementia, family carer, refusals of care, resistance‐to‐care

## Abstract

**Background:**

People with dementia sometimes refuse assistance with personal care activities such as washing or dressing. We aimed to investigate the factors associated with refusals of care in advanced dementia.

**Methods:**

A cross‐sectional study using informant‐based measures. Participants were people with advanced dementia and their caregivers (family carers or care‐home staff) (*n* = 260, 130 dyads) in the UK. Mixed effects linear models were used to examine the effects of neuropsychiatric behaviours, ability with activities of daily living, professional input, co‐morbidities, psychotropic medications, environment modifications, and caregiver factors including type and training status on refusals of care. The Refusal of Care Informant Scale was used, range 1–13; higher scores indicate more refusal behaviours.

**Results:**

Higher independence in activities of daily living was associated with less refusal behaviours (coefficient = −0.11, *p* < 0.001 [95% confidence interval −0.15, −0.07]). Higher agitation was associated with more refusal behaviours (0.11, *p* < 0.001 [0.06, 0.15]). No other statistically significant differences were found. There was no demonstrable evidence of differences in number of refusals of care between family and care‐home caregivers or between dementia‐trained or ‐untrained caregivers.

**Conclusions:**

Results suggest refusals of care have similar prevalence regardless of caregiver type (family or care home) or dementia training status, indicating that current dementia training has no impact on refusals of care or may not be implemented as intended. Improving independence in activities of daily living and reducing agitations may help prevent refusals of care. To establish causality, future research should consider embedding these factors into interventions targeting refusal of care.

## Key points

- Refusals of care occur when a person with dementia finds assistance with their basic activities of daily living, such as washing or dressing, unwelcome and demonstrates in some way that they are not happy to receive help.
- In advanced dementia, higher independence in activities of daily living was associated with less refusals and higher agitation was associated with more refusals.
- Caregiver setting (family or care home) or training in dementia (none or some) were not associated with refusals of care.

## 1 INTRODUCTION

There are over 55 million people living with dementia globally,^1^ with over 885,000 in the UK.^2^ In the UK, 60% of people with dementia are in the advanced stages,^2^ which can last up to 5 years.^3^ In the advanced stages, people living with dementia require considerable assistance with basic activities of daily living such as going to the toilet, washing, and dressing.^4^ Often this assistance with personal care is provided by family carers or care‐home staff (henceforth collectively termed as caregivers).^2^ Sometimes receiving personal care assistance is not acceptable to the person with dementia and they refuse it.^5^, ^6^, ^7^, ^8^

Refusals of care can be distressing for both the person with dementia^9^ and their caregiver. For example, family carers have been found to experience psychological distress as refusals provide barriers to completing functional care.^10^ Whereas care‐home staff can experience feelings of discomfort created by the tension between their caring values and the situations refusals generate.^11^ Refusals can lead to caregiver stress and burden^6^, ^12^ and have potential negative consequences such as urine burns if adequate care is not completed.^13^ Interventions evidenced as reducing refusals of care include playing music during care or using different bathing techniques such as a thermal bath or strip wash.^14^ Refusals of care have been linked to factors such as the caregiver's communication style^12^, ^15^; uninvited direct care,^16^ psychotic symptoms such as hallucinations and delusions^17^, ^18^; depression^8^, ^17^, ^18^; the person not understanding, being confused or disoriented,^17^, ^19^ or inadequate staffing, workload, and time management in care homes.^19^ However, there has been limited research investigating factors in combination to identify those associated with refusals of care in dementia. Factors can be categorised as, those related to the:

- Person with dementia: dementia sub‐type, age, gender, ethnicity, psychotropic medication use, ability in activities of daily living, neuropsychiatric behaviours, co‐morbidities, health status.
- Caregiver: caregiver type, training, confidence, support needs, management style.
- Environment: modifications to the bedroom and/or bathroom, professional involvement.

Better understanding of the factors associated with refusals of care in advanced dementia should illuminate the key areas to target to reduce refusals.

In this study, we aimed to examine the factors listed above to determine those associated with refusals of assistance with personal care in advanced dementia in both family (informal) and care‐home (formal) settings. We hypothesised that there would be differences in refusals of care between care settings (care home and family) and between caregivers with dementia training and those without (some family caregivers access dementia training and some care‐home staff do not). We theorised: (1) that as formal care staff in care homes were professional caregivers they would experience less refusals of care, and (2) that trained caregivers, irrespective of care setting, would experience less refusals of care.

Ethical approval was sought and given from the Queen's Square Research Ethics Committee, London (reference: 251339).

## 2 METHODS

### 2.1 Study design and participants

A cross‐sectional study using informant‐based measures was used.

Participants were recruited in dyads: a person with advanced dementia either supported at home or in a care home, aged 65 or over, receiving physical assistance with personal care activities such as washing or dressing and the family carer or care‐home staff member who regularly assisted them. Family carers were the primary caregiver for the person with dementia and were over the age of 18. Care‐home staff were usually assisting the person with dementia with their care at least six instances a week and were over the age of 18.

To determine the eligibility criteria of advanced dementia, the Frontier Dementia Rating Scale (FRS),^20^ was used after consent, but before any other data collection. The FRS is a well validated 30‐item dementia staging tool, completed through a caregiver interview, assessing changes in areas such as household chores, selfcare and behaviour.

### 2.2 Recruitment

People with dementia in England not living in care homes and their family carers were identified from either a local research‐team database or a national database called Join Dementia Research, where those registered had expressed an interest in being contacted about research opportunities. Written information was provided, and the first author discussed the research with potential participants.

Care homes in the UK offer 24‐h accommodation, meals, and assistance with personal care either with or without qualified nursing staff. Care homes assisting people with dementia in the East of England were identified from the Care Quality Commission^21^ database in the public domain. After a letter, follow‐up telephone call and visit from the first author, eight care‐home managers agreed for their care homes (two with qualified nursing and six without qualified nursing) to be involved in the study. Written information was provided to residents, residents' family members and care staff identified by managers as eligible and who might be interested. The first author was available to discuss the research.

Written consent or advice was obtained for all participants. For people with dementia assessed as lacking the capacity to decide about whether to take part, in line with the Mental Capacity Act of England and Wales 2005,^22^ their family members or close friends provided written advice as to whether they would have been likely to decide to take part if they had capacity.

### 2.3 Sample size calculation

Sample size was calculated a priori using GPower.^23^ Effect sizes were not available for the dependent variable (Refusal of Care Informant Scale [RoCIS]).^7^ Therefore, we estimated the sample size assuming a moderate effect size (Cohen's *d*) of 0.5, for the difference of refusals between care‐home staff and family carers. A minimum sample of 128 dyads would detect a medium effect at 5% (two‐sided) level of significance with 80% power.

### 2.4 Data collection and measurements

Data were collected in England between January 2019 and May 2021. Data collection was predominantly informant‐based and took place either face‐to‐face, over the telephone or online via Zoom with caregivers. Face‐to‐face data collection took place in family homes and in the care homes where the care‐home staff worked. Caregivers answered the first author's questions about the person with dementia and also filled in three self‐complete questionnaires about themselves (returned to the first author directly or via a pre‐paid postal envelope). Dementia sub‐type was determined from people with dementia's General Practitioners (GPs). Consent to contact GPs for diagnoses was obtained at the time of study consent.

#### 2.4.1 Measures

**Refusals of care** were assessed through the RoCIS,^7^ a 13‐item, questionnaire developed and validated as part of the first author's programme of research^7^ asking whether refusal behaviours such as verbal refusals, clamping the jaw or pushing the caregiver away were present during personal care interactions in the last month (higher scores indicate more refusal behaviours).

**Caregiver type (care home or family) and dementia training (yes, no)** were assessed with a bespoke questionnaire; these two factors were our hypothesised main exposures of interest.

**Care setting** was a grouping variable—either a care home or the family setting for each dyad. Each care home was defined as a cluster and all dyads from the family setting were assigned to one (family) cluster.

**Demographic details** such as age, gender and ethnicity, and psychotropic medication use and health status over the last month were taken using a bespoke questionnaire.

**Neuropsychiatric symptoms** were assessed using the 12‐domain Neuropsychiatric Inventory (NPI) Questionnaire (NPI for family carers, NPI nursing home version for care‐home staff [NPI‐NH]).^24^, ^25^ The domains constitute items such as anxiety, delusions and disinhibition and are rated by frequency and severity. Total frequency × severity score (0–144) was used, with higher scores indicating higher presence of neuropsychiatric symptoms.

**Agitation** was assessed using the Cohen Mansfield Agitation Inventory (CMAI),^26^ a 29‐item measure including items such as screaming, biting, and pushing. Total score was used (29–203), with higher scores indicating higher presence of agitation.

**Functional ability** was assessed using the Alzheimer's Disease Cooperative Study Activities of Daily Living Inventory for severe dementia (ADCS‐ADLsev19).^27^, ^28^ A 19‐item questionnaire scored out of 54 (higher scores relate to more independence) asking whether certain daily activities such as bathing, getting dressed or going out were performed independently or with different levels of supervision or assistance.

**Environmental modifications** were assessed using a reduced version of the Home Environmental Assessment Protocol for Dementia (HEAP) to determine the presence or absence of bedroom or bathroom modifications.^29^

**Professional input,** including any GP, nurse, medical, allied health professional, or hospital visit, in the last 3 months was assessed using a reduced version of the Client Service Receipt Inventory^30^ to determine presence or absence.

**Comorbidities** such as liver disease, heart failure and cancer were assessed using the Charlson Comorbidity 19‐item Index.^31^ Total score was used with higher score indicating more comorbidities.

**Caregiver confidence and training needs** were assessed using a bespoke self‐report questionnaire assessing confidence and perceived training needs in relation to different personal care activities. Two scores were calculated separately, with higher scores indicating more confidence or higher training needs.

**Caregiver management style** was assessed using the 28‐item caregiver self‐report Dementia Management Strategies Scale^32^ which assesses caregiver management styles (criticism, active management, and encouragement) used over the previous month. A slightly adapted version was used changing the term ‘my relative’ to ‘the person’. For this study, scores of each style were converted to percentages and the predominant management style coded for each caregiver.

### 2.5 Analysis

Analysis was conducted in Stata17.^33^

#### 2.5.1 Descriptive analysis

Mean and standard deviation (SD) were calculated for continuous and normally distributed variables, and median and interquartile range (IQR) for continuous but skewed data, and frequency and percentage for categorical variables. Effect sizes were calculated for both caregiver type and caregiver training (our main exposures of interest) by running separate *t*‐tests with refusals of care and dividing the mean difference by the overall SD of the difference.^34^

#### 2.5.2 Linear mixed model analysis

Mixed effects linear models were used for investigating the factors associated with refusals of care. The models included a random intercept for care setting to account for the variation between care settings and any potential intra‐cluster correlation between participants from the same care setting (nested data).^35^, ^36^ To screen and select predictor variables for inclusion in the final model, univariable mixed effects linear regression models were fitted for each predictor with random intercept for care setting. Each of these models included refusal of care as the outcome variable, and one predictor variable from the set of predictors: caregiver type, caregiver training, dementia sub‐type, age, gender, ethnicity, psychotropic medication use, health status, activities of daily living ability, neuropsychiatric behaviours, co‐morbidities, environment modifications, professional involvement, caregiver confidence, caregiver support needs, or caregiver management style. Predictor variables with *p*‐values for the regression coefficients higher than 25% (*p* > 0.25) were not considered to be potentially important predictors of refusal of care and were not included in the multivariable model.^37^

The remaining predictor variables, those with *p* < 0.25 in the univariable models, were included together in a multivariable mixed effects linear regression model. Subsequently, independent variables showing very weak associations (*p* ≥ 0.90) in the multivariable model were excluded, and the final model re‐estimated.

The final multivariable model was used for regression diagnostics and model checking. The distribution of random effects and overall residuals were examined via Q‐Q plot and boxplot for assessing normality. Variance inflation factors were examined to check for multicollinearity.

## 3 RESULTS

### 3.1 Descriptive statistics

Table 1 shows descriptive statistics of the 130 dyads (260 participants) who took part in the study: 106 from family settings and 24 from care‐home settings. The outcome measure, refusals of care, was available on 129 dyads. The distribution of age of people with dementia was approximately normal with mean 80 years and SD 7.94 years. The distribution of caregivers age was skewed with median 70 years and IQR 58–76 years, 51.54% of people with dementia and 29.23% of caregivers were male. People with dementia and caregivers were predominantly White British (96.92% and 96.12% respectively). Alzheimer's disease was the most common subtype of dementia (44.62%), with 20.77% mixed dementia and 15.38% vascular dementia.

**TABLE 1:** Summary of participant characteristics

	Refusals of care reported *n* = 88 dyads	No refusals of care reported *n* = 41 dyads	Total *n* = 130 dyads
Age			
Person with dementia mean (SD), range	80.48 (8.17), 65–99	79.37 (7.31), 65–94	80.22 (7.94), 65–99
Caregiver ^a^median (IQR), range	69.5 (55.5–76), 19–87	72 (64–79), 23–87	70 (58–76), 19–87
Gender male *n* (%)			
Person with dementia	46 (52.27)	21 (51.22)	67 (51.54)
Caregiver	26 (29.55)	12 (29.27)	38 (29.23)
Ethnicity *n* (%)			
Person with dementia			
White	84 (95.45)	41 (100)	126 (96.92)
Black	1 (1.14)	0 (0)	1 (0.77)
Asian	2 (2.27)	0 (0)	2 (1.54)
Mixed	1 (1.14)	0 (0)	1 (0.77)
Caregiver			
White	83 (95.40)	40 (97.56)	124 (96.12)
Black	1 (1.15)	1 (2.44)	2 (1.55)
Asian	3 (3.45)	0 (0)	3 (2.33)
Mixed	0 (0)	0 (0)	0 (0)
Dementia subtype *n* (%)			
Alzheimer's disease	37 (42.05)	20 (48.78)	58 (44.62)
Mixed dementia	21 (23.86)	6 (14.63)	27 (20.77)
Vascular dementia	12 (13.64)	8 (19.51)	20 (15.38)
Dementia Lewy body	7 (7.95)	2 (4.88)	9 (6.92)
Frontal temporal dementia	4 (4.55)	1 (2.44)	5 (3.85)
Other	3 (3.41)	4 (9.76)	7 (5.38)
Unknown	4 (4.55)	0 (0)	4 (3.08)
Refusals of care (RoCIS score), mean (SD), range	4.36 (3.37), 1–13	0 (0), 0–0	2.98 (3.45), 0–13
Caregiver type, family *n* (%)	66 (75)	39 (95.12)	105 (81.41)
Caregiver training, yes *n* (%)	42 (47.73)	9 (21.95)	51 (39.53)
Neuropsychiatric symptoms (NPI 12‐item score), mean (SD), range	124.51 (172.78), 1–1131	64.32 (79.23), 0–299	105.38 (151.81), 0–1131
Agitation (CMAI score), mean (SD), range	47.14 (13.71), 29–85	37.10 (9.55), 29–65	43.92 (13.34), 29–85
ADLs (ADCS‐ADLsev19 score), mean (SD), range	17.09 (12.76), 0–46	27.15 (9.07), 6–43	20.31 (12.58), 0–46
Psychotropic medication, yes *n* (%)	54 (62.07)	23 (56.10)	77 (60.16)
Health status, usual self *n* (%)	70 (79.55)	38 (92.68)	108 (83.72)
Comorbidities (Charlson Comorbidity Index), mean (SD), range	2.90 (1.60), 1–8	3 (1.70), 1–7	2.93 (1.65), 1–8
Environment modifications, yes *n* (%)	44 (50.57)	16 (39.02)	60 (46.88)
Professional involvement, yes *n* (%)	74 (85.06)	32 (78.05)	106 (82.81)
Caregiver confidence, mean (SD), range	52.77 (15.34), 0–70	46.61 (16.72), 9–70	50.81 (15.99), 0–70
Caregiver support needs, mean (SD), range	3.13 (4.12), 0–15	2.71 (4.18), 0–15	2.99 (4.13), 0–15
Caregiver management style (DMSS), *n* (%)			
Active management	72 (81.82)	29 (70.73)	101 (78.29)
Encouragement	10 (11.36)	10 (24.39)	20 (15.50)
Criticism	1 (1.14)	0 (0)	1 (0.78)
Active management/encouragement	5 (5.68)	2 (4.88)	7 (5.43)

Abbreviations: ADCS‐ADLsev19, Alzheimer's Disease Cooperative Study Activities of Daily Living Inventory for severe dementia; CMAI, Cohen Mansfield Agitation Inventory; DMSS, Dementia Management Strategies Scale; IQR, interquartile range; NPI, Neuropsychiatric Inventory; RoCIS, Refusal of Care Informant Scale; SD, standard deviation.

^a^ Median and IQR used for caregiver age as the variable was not distributed normally.

### 3.2 Linear mixed model analysis

Screening via univariate linear mixed models suggested that dementia sub‐type, gender, ethnicity, and caregiver management may not be important predictors of refusals of care (all *p* > 0.25), so these variables were excluded (see Supporting Information A) from the subsequent analysis using a multivariable model. Caregiver type (family or care home) and caregiver training (no, yes)—the main hypothesised predictors of interest, also showed no effect on refusals of care (0.23, *p* = 0.764 [95% confidence interval −1.29, 1.76] and 0.30, *p* = 0.630 [−0.91, 1.51] respectively). However, these variables were directly linked to our hypotheses of interest so were retained in the final model.

An initial multivariable model was fitted (see Supporting Information B) which showed caregiver confidence and person with dementia health status as potentially the weakest predictors of refusal of care (*p* > 0.90), so these were removed from the final model.

**FIGURE 1:**
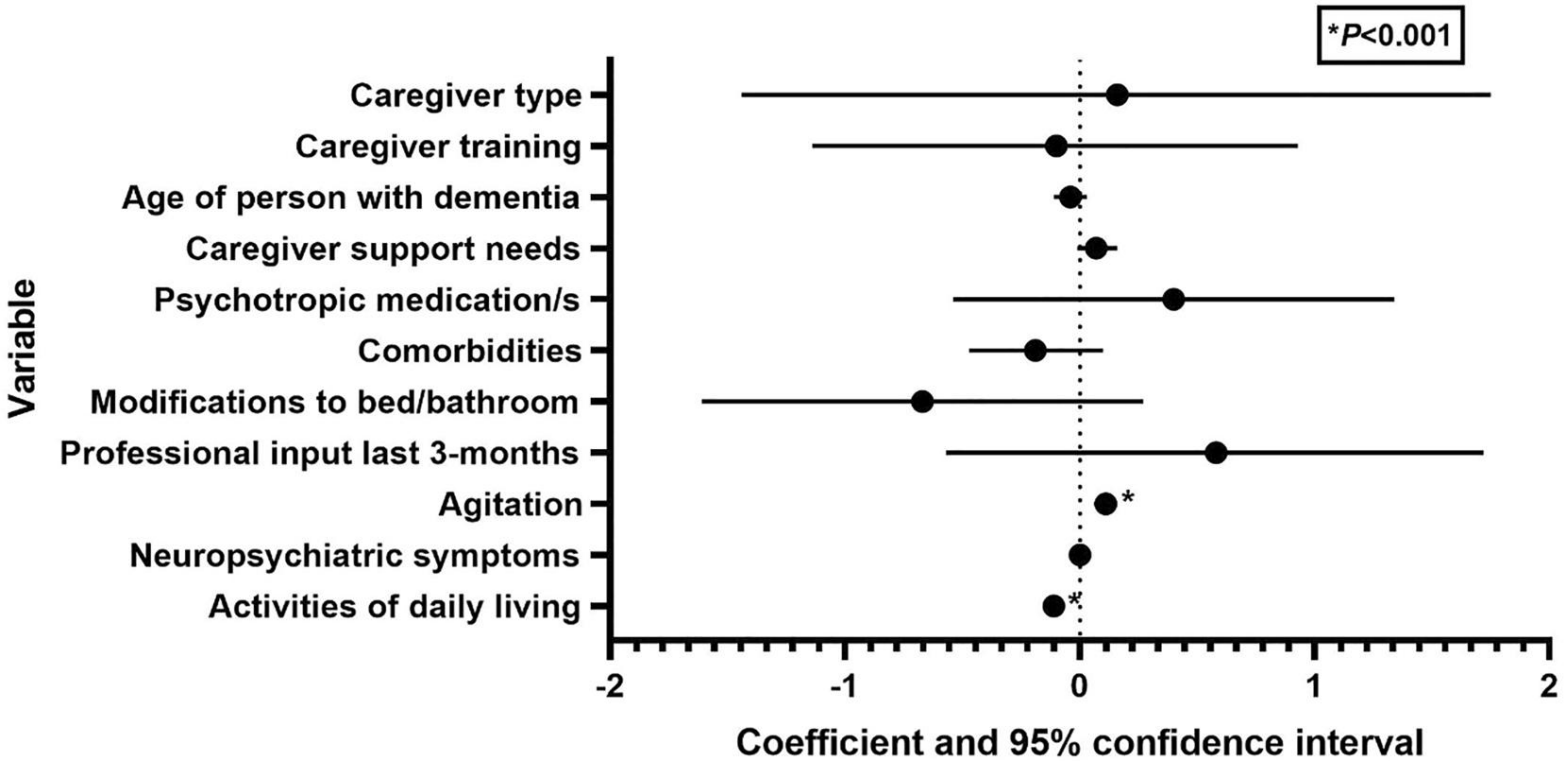
Forest plot of adjusted multivariate model variable coefficients and 95% confidence intervals.

Table 2 shows the unadjusted as well as adjusted coefficients and the statistical inferences for variables included in the final multivariable. Higher independence in ADLs was associated with less refusal behaviours (−0.11, *p* < 0.001 [−0.15, −0.07]). Higher levels of agitation were associated with more refusal behaviours (0.11, *p* < 0.001 [0.06, 0.15]). No associations were found between refusals of care and age of the person with dementia, caregiver support needs, psychotropic medication use, comorbidities, modifications to the bathroom or bedroom environment, professional input within the last 3 months, or neuropsychiatric symptoms as assessed by the NPI. The differences in refusals between caregiver type (care home or family) (Cohen's *d* = 0.068) or between caregivers with or without dementia training (*d* = 0.086) were small and the effect sizes do not appear to be clinically meaningful. A visual representation of the adjusted coefficients and the associated 95% confidence intervals is given in Figure 1.

**TABLE 2:** Results from the mixed effect linear regression

Variable	Unadjusted (univariable models)			Adjusted (multivariable model)		
	Coefficient (SE)	*p*‐value	95% conf. Interval	Coefficient (SE)	*p*‐value	95% conf. Interval
Caregiver type (^a^Family)	0.23 (0.78)	0.764	−1.29, 1.76	0.16 (0.81)	0.848	−1.44, 1.75
Caregiver training (^a^No)	0.30 (0.62)	0.630	−0.91, 1.51	−0.10 (0.53)	0.843	−1.14, 0.93
Age of person with dementia	−0.07 (0.04)	0.057	−0.15, 0.00	−0.04 (0.04)	0.246	−0.11, 0.03
Caregiver support needs	0.16 (0.05)	0.003	0.06, 0.27	0.07 (0.04)	0.101	−0.01, 0.16
Psychotropic (^a^No)	1.38 (0.61)	0.023	0.19, 2.57	0.40 (0.48)	0.408	−0.54, 1.34
Comorbidities (Charlson Comorbidity Index)	−0.28 (0.18)	0.130	−0.63, 0.08	−0.19 (0.14)	0.198	−0.47, 0.10
Modifications to bed/bathroom (^a^No)	0.86 (0.60)	0.155	−0.33, 2.04	−0.67 (0.48)	0.165	−1.61, 0.27
Professional input last 3‐months (^a^No)	0.98 (0.80)	0.221	−0.59, 2.54	0.58 (0.58)	0.322	−0.57, 1.72
Agitation (CMAI)	0.15 (0.02)	0.000	0.11, 0.18	0.11 (0.02)	0.000	0.06, 0.15
Neuropsychiatric symptoms (NPI 12‐item)	0.01 (0.00)	0.000	0.01, 0.01	0.00 (0.00)	0.277	−0.00, 0.01
ADLs (ADCS‐ADLsev19)	−0.12 (0.02)	0.000	−0.17, −0.08	−0.11 (0.02)	0.000	−0.15, −0.07

Abbreviations: ADCS‐ADLsev19, Alzheimer's Disease Cooperative Study Activities of Daily Living Inventory for severe dementia; CMAI, Cohen Mansfield Agitation Inventory; NPI, Neuropsychiatric Inventory; SE, standard error.

^a^ Reference category.

Variance inflation factors for variables in the final model were checked for multicollinearity and were all <5, ranging between 2.21 for care setting and 1.06 for professional input in the last 3 months (mean 1.51), showing no obvious concern about collinearity (see Supporting Information C). An assessment of the distribution of overall residuals from the adjusted model showed satisfactory compliance with normal distribution (see Supporting Information D). Distribution of random effects were assessed using Q‐Q plot however since there were only nine groups it was difficult to interpret normality from this plot (see Supporting Information E).

## 4 DISCUSSION

While multiple studies have developed and assessed interventions for refusals of care,^14^, ^38^ this is a novel study determining the factors associated with refusals of care in advanced dementia. Results show that greater dependence in ADLs and greater level of agitation are associated with refusals of care in advanced dementia. Contrary to expectation, we did not find a difference on refusals of care between professional care‐home staff and family carers. Therefore, people with dementia did not respond better to care from a familiar family carer or as theorised, from an experienced professional caregiver. There was also no difference between trained and untrained caregivers (those who had received, or had not received, dementia training). These results indicate that refusals of care have similar prevalence regardless of caregiver status. This has implications for the effects of dementia training as an intervention to prevent or reduce refusals of care. It may be that dementia training does not cover aspects that would influence the occurrence of refusals of care, that training is not put into effect, or that training is not effective, for example, it did not channel efficacious features such as individual sessions, active participation or learning support.^39^

The results showed that neuropsychiatric symptoms measured by the NPI had no association with refusals of care, yet those in the CMAI did. Although these two measures both focus on neuropsychiatric symptoms, they measure different things. The NPI assesses psychotic symptoms (hallucinations and delusions), behaviours such as depression and aggression, and sleep and eating disorders in one questionnaire,^24^, ^25^ whereas the CMAI focusses on visible expressions of agitation such as pacing, hiding things, and screaming.^26^ Due to the differences in focus, the NPI is likely to be less sensitive in discerning changes in agitation in comparison to the CMAI.^40^

Although our analysis identified an association between agitation and refusals of care in advanced dementia, the underlying causes of that agitation are varied and not always avoidable. For instance, the person may have been in pain or have had unmet needs; it is also likely that agitation was a result of the severity of the dementia itself.^41^ Non‐pharmacological interventions and approaches have been found to reduce agitation in care‐home settings. These include person‐centred care, communication skills training, adapted dementia care mapping, activities, music therapy by protocol, and sensory intervention.^42^ Implementing these could be first steps to reducing agitation. It is also important to acknowledge that some agitation may not be modifiable, and expectations need to be kept in check. Further research is needed to focus on family settings to see what works to reduce agitation there,^42^ where possible.

Often, refusals of care have been conflated with other behavioural expressions such as agitation or aggression in dementia, for example, in assessment scales of behavioural change.^43^ However, work examining refusals and other behavioural expressions in dementia has shown that refusals are separate entities to agitation or aggression, caused by different contributory factors and thus require differently focussed interventions.^44^, ^45^, ^46^ Refusals of care occur within the context of care interactions,^47^ therefore it is not surprising that more dependence in ADLs is associated with refusals, since more care interactions would be required to assist the person with these activities as they become more dependent. ADL dependence would also be a clear sign of greater cognitive deficits, that is, reflecting a person's difficulties in processing what is occurring around them.

That independence with ADLs was associated with less refusal of care behaviours may indicate that refusals increase as dementia becomes more advanced. Or that as impairment increases, a lack of control and ability to conduct care activities in the ways in which the person with dementia could understand or would want them to be conducted leads to refusals. Feelings of dependence may contribute to the person with dementia becoming frustrated leading to refusals. Additionally, increased assistance required with ADLs, equates to more care interactions with caregivers, which would increase the assistance time in which a person may refuse assistance. Caregivers' actions can influence refusals^15^, ^16^ and refusals can negatively affect caregiver wellbeing.^6^ Therefore, as a person with dementia's dependence increases it is paramount that their caregiver(s) are equipped with the necessary skills to reduce and manage refusals. Our results point to two key factors for interventions aimed at refusals of care to consider: agitation and dependence in ADLs.

### 4.1 Strengths, limitations, and suggestions for future research

This study provides insight into the factors associated with refusals of care in advanced dementia, indicating targets for intervention as part of their prevention and management. Limitations include being reliant on subjective informant reported measures of refusals of care, having less data from care‐home settings in comparison to family settings. We used the CMAI and NPI total scores in our analysis and have not assessed individual or grouped behaviours/domains, where differences may have been found. Although we assessed multiple factors, we did not assess pain or delirium, which may have been relevant factors associated with refusals. We also did not examine care interactions, which could be one way to provide insightful information about factors associated with refusals of care. Future research should work to develop suitable interventions to reduce agitation and optimise as much independence with ADLs as possible in advanced dementia. Additionally, examining other possible factors (for example, pain or unmet needs) related to refusals of care in statistical models would improve knowledge.

## 5 CONCLUSIONS AND IMPLICATIONS FOR PRACTICE

Agitation and dependence in ADLs were associated with refusals of care in advanced dementia. Importantly, there were no associations with care setting (family or care home) or caregiver dementia training (yes or no) and refusals of care, indicating that refusals of care have similar prevalence regardless of caregiver status. Additionally, there are possible implications for the effects of dementia training as an intervention to prevent or reduce refusals of care. It may be that current dementia training does not cover aspects that would influence the occurrence of refusals of care, that dementia training is not effectively delivered, or that training is not put into practice. Future research should determine key components to reduce refusals, including exploring the value of reducing agitation and maximising independence.

## CONFLICT OF INTEREST

The authors have no conflict of interest to declare.

## Supporting information

Supporting Information S1

## Data Availability

The data that support the findings of this study are available on request from the corresponding author. The data are not publicly available due to privacy or ethical restrictions.
