# Supplementary material for: What are the factors associated with people with advanced dementia refusing assistance with personal care?
Source: Int J Geriatr Psychiatry. 2022 Dec 9;38(1):e5857. doi: 10.1002/gps.5857 (PMC10107826; doi:10.1002/gps.5857)
Supplement: Supplementary file 1 — Supporting Information S1 [file GPS-38-0-s001.docx]

**SUPPLEMENTARY MATERIAL A**

| **Table A: Univariate Mixed Effects Linear Models for Screening Independent Variables** | | | |
| --- | --- | --- | --- |
|  | **Unadjusted** | | |
| **Independent variable** | **Coefficient (SE)** | ***P* =z** | **95% Conf. Interval** |
| Caregiver type (*Family) | 0.23 (0.78) | 0.764 | (-1.29, 1.76) |
| Diagnosis (*AD)  -Non-AD  -Unknown | -0.32 (0.62)  -0.41 (1.77) | 0.604  0.819 | (-1.53, 0.89)  (-3.88, 3.07) |
| Female (*Male) | 0.01 (0.61) | 0.982 | (-1.17, 1.11) |
| Ethnicity/BAME (*White) | -0.49 (1.74) | 0.778 | (-3.91, 2.93) |
| Age of person with dementia | -0.07 (0.04) | 0.057 | (-0.15, 0.00) |
| Caregiver support needs | 0.16 (0.05) | 0.003 | (0.06, 0.27) |
| Caregiver confidence | 0.03 (0.20) | 0.140 | (-0.01, 0.07) |
| Psychotropic (*No) | 1.38 (0.61) | 0.023 | (0.19, 2.57) |
| Caregiver training (*No) | 0.30 (0.62) | 0.630 | (-0.91, 1.51) |
| Health status usual for person (*No) | -1.11 (0.81) | 0.173 | (-2.70, 0.49) |
| Charlson Comorbidity Index | -0.28 (0.18) | 0.130 | (-0.63, 0.08) |
| Modifications to bed/bathroom (*No) | 0.86 (0.60) | 0.155 | (-0.33, 2.04) |
| Professional input last 3-months (*No) | 0.98 (0.80) | 0.221 | (-0.59, 2.54) |
| CMAI | 0.15 (0.02) | 0.000 | (0.11, 0.18) |
| NPI 12-item | 0.01 (0.00) | 0.000 | (0.01, 0.01) |
| ADLSEV | -0.12 (0.02) | 0.000 | (-0.17, -0.08) |
| DMSS dominant trait (*Active management)  -Encouragement  -Criticism  -Active management/encouragement | -0.66 (0.84)  2.94 (3.43)  -0.06 (1.34) | 0.430  0.392  0.964 | (-2.30, 0.98)  (-3.79, 9.67)  (-2.68, 2.56) |
| *: Reference category; SE: Standard Error; CMAI: Cohen Mansfield Agitation Inventory; NPI: Neuropsychiatric Inventory; ADCS-ADLsev19: Alzheimer’s Disease Cooperative Study Activities of Daily Living Inventory for severe dementia | | | |

**SUPPLEMENTARY MATERIAL B**

| **Table B: Results of Initial Adjusted Mixed Effects Linear Regression Model** | | | | | | |
| --- | --- | --- | --- | --- | --- | --- |
| Variable | **Unadjusted (univariate models)** | | | **Adjusted (multi** | | |
|  | **Coefficient**  **(SE)** | ***P* =z** | **95% Conf. interval** | **Coefficient**  **(SE)** | ***P* =z** | **95% Conf. interval** |
| Care home (*Family setting) | 0.23 (0.78) | 0.764 | (-1.29, 1.76) | 0.16 (0.82) | 0.851 | (-1.46, 1.77) |
| Caregiver training (*No) | 0.30 (0.62) | 0.630 | (-0.91, 1.51) | -0.12 (0.53) | 0.828 | (-1.16, 0.93) |
| Age of person with dementia | -0.07 (0.04) | 0.057 | (-0.15, 0.02) | -0.04 (0.04) | 0.247 | (-0.11, 0.03) |
| Caregiver support needs | 0.16 (0.05) | 0.003 | (0.06, 0.27) | 0.07 (0.04) | 0.101 | (-0.01, 0.16) |
| Caregiver confidence | 0.03 (0.02) | 0.140 | (-0.01, 0.07) | 0.00 (0.02) | 0.973 | (-0.03, 0.03) |
| Psychotropic (*No) | 1.38 (0.61) | 0.023 | (0.19, 2.57) | 0.40 (0.48) | 0.411 | (-0.55, 1.35) |
| Health status usual for person | -1.11 (0.81) | 0.173 | (-2.70, 0.49) | -0.07 (0.62) | 0.909 | (-1.29, 1.15) |
| Charlson Comorbidity Index | -0.28 (0.18) | 0.130 | (-0.63, 0.08) | -0.19 (0.14) | 0.197 | (-0.47, 0.10) |
| Modifications to bed/bathroom (*No) | 0.86 (0.60) | 0.155 | (-0.33, 2.04) | -0.66 (0.49) | 0.179 | (-1.63, 0.30) |
| Professional input last 3-months (*No) | 0.98 (0.80) | 0.221 | (-0.59, 2.54) | 0.57 (0.59) | 0.334 | (-0.58, 1.72) |
| CMAI | 0.15 (0.02) | 0.000 | (0.11, 0.18) | 0.11 (0.02) | 0.000 | (0.06, 0.15) |
| NPI 12-item | 0.01 (0.00) | 0.000 | (0.01, 0.01) | 0.00 (0.00) | 0.284 | (-0.00, 0.01) |
| ADLSEV | -0.12 (0.02) | 0.000 | (-0.17, -0.08) | -0.11 (0.02) | 0.000 | (-0.15, -0.07) |
| *: Reference category; SE: Standard Error; CMAI: Cohen Mansfield Agitation Inventory; NPI: Neuropsychiatric Inventory; ADCS-ADLsev19: Alzheimer’s Disease Cooperative Study Activities of Daily Living Inventory for severe dementia | | | | | | |

**SUPPLEMENTARY MATERIAL C**

| **Table C: Variation Inflation Factors (VIF)** | | |
| --- | --- | --- |
| **Variable** | **VIF** | **1/VIF** |
| Care home (*Family setting) | 2.21 | 0.45 |
| CMAI | 2.11 | 0.47 |
| NPI 12-item | 1.99 | 0.50 |
| Age of person with dementia | 1.66 | 0.60 |
| Caregiver training (*No) | 1.45 | 0.69 |
| Modifications to bed/bathroom (*No) | 1.26 | 0.80 |
| ADLSEV | 1.26 | 0.80 |
| Charlson Comorbidity Index | 1.24 | 0.81 |
| Psychotropic (*No) | 1.21 | 0.82 |
| Caregiver support needs | 1.17 | 0.86 |
| Professional input last 3-months (*No) | 1.06 | 0.94 |
| **Mean VIF** | 1.51 |  |
| *: Reference category; CMAI: Cohen Mansfield Agitation Inventory; NPI: Neuropsychiatric Inventory; ADCS-ADLsev19: Alzheimer’s Disease Cooperative Study Activities of Daily Living Inventory for severe dementia | | |

**SUPPLEMENTARY MATERIAL D**


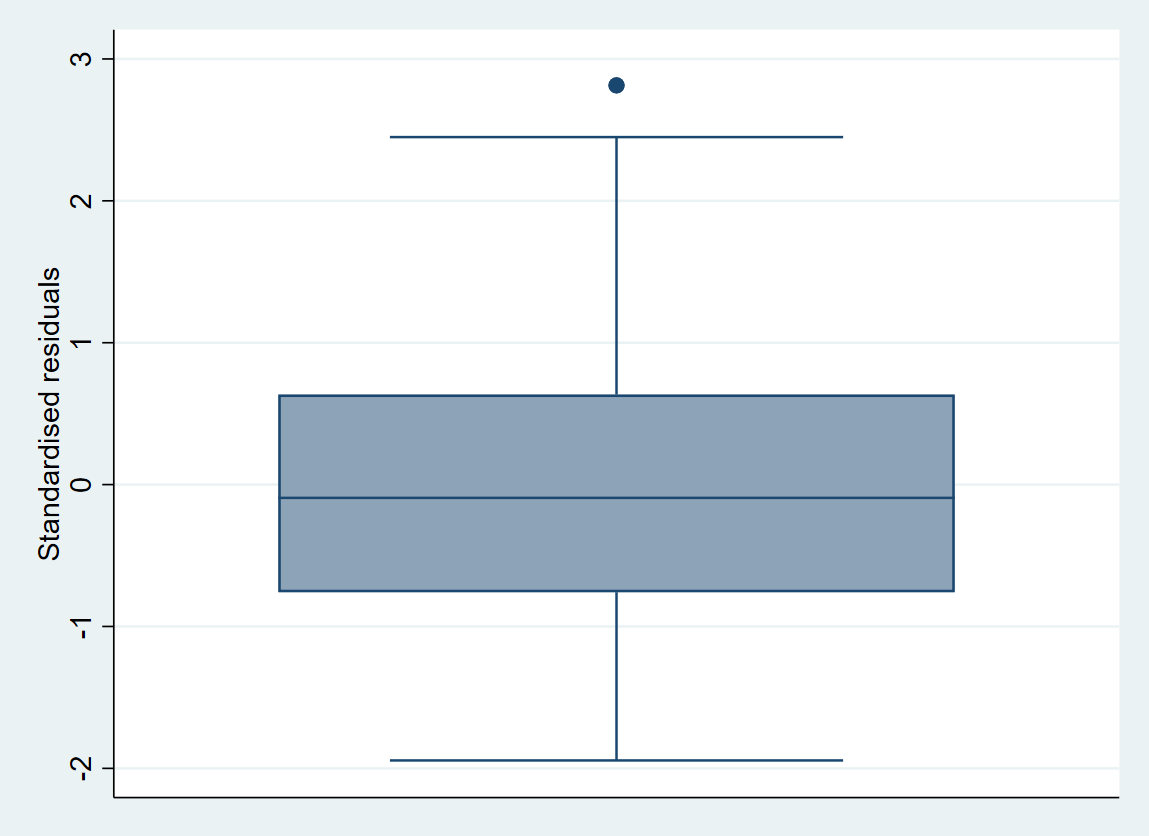


Figure 1: Box plot of standardised residuals


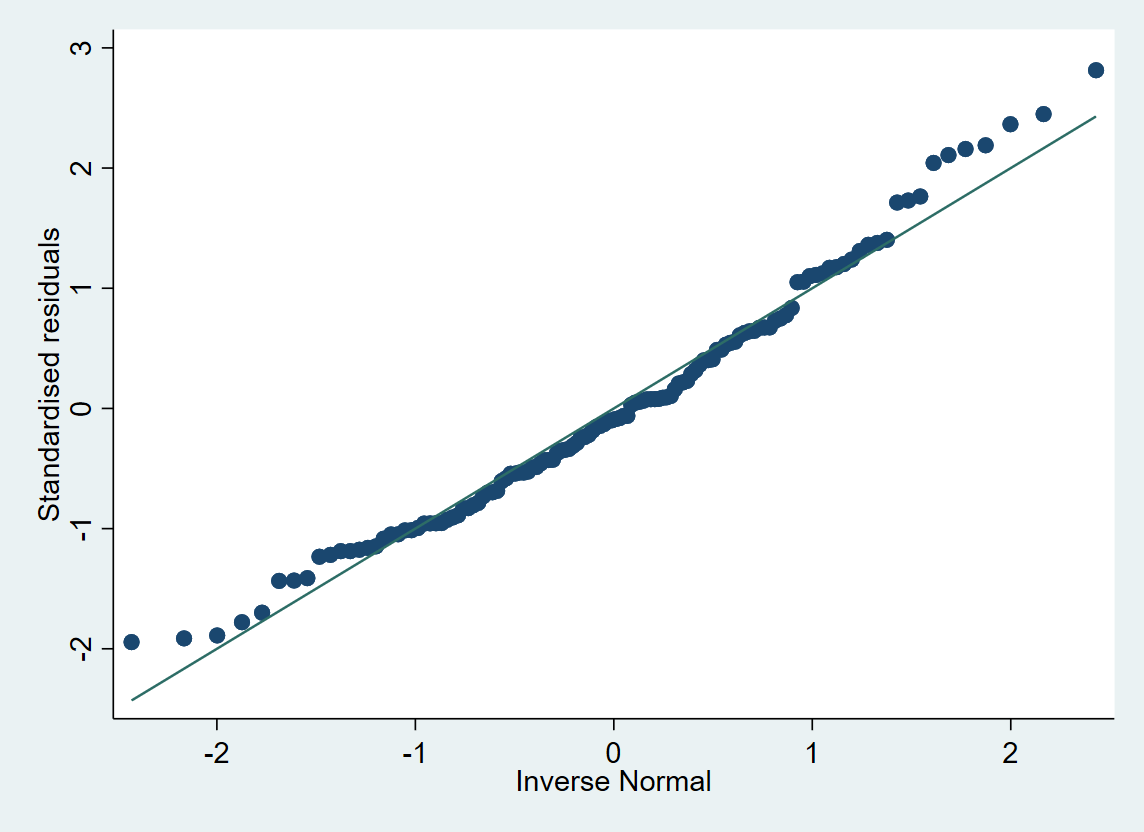


Figure 2: QQ plot of standardised residuals

**SUPPLEMENTARY MATERIAL E**


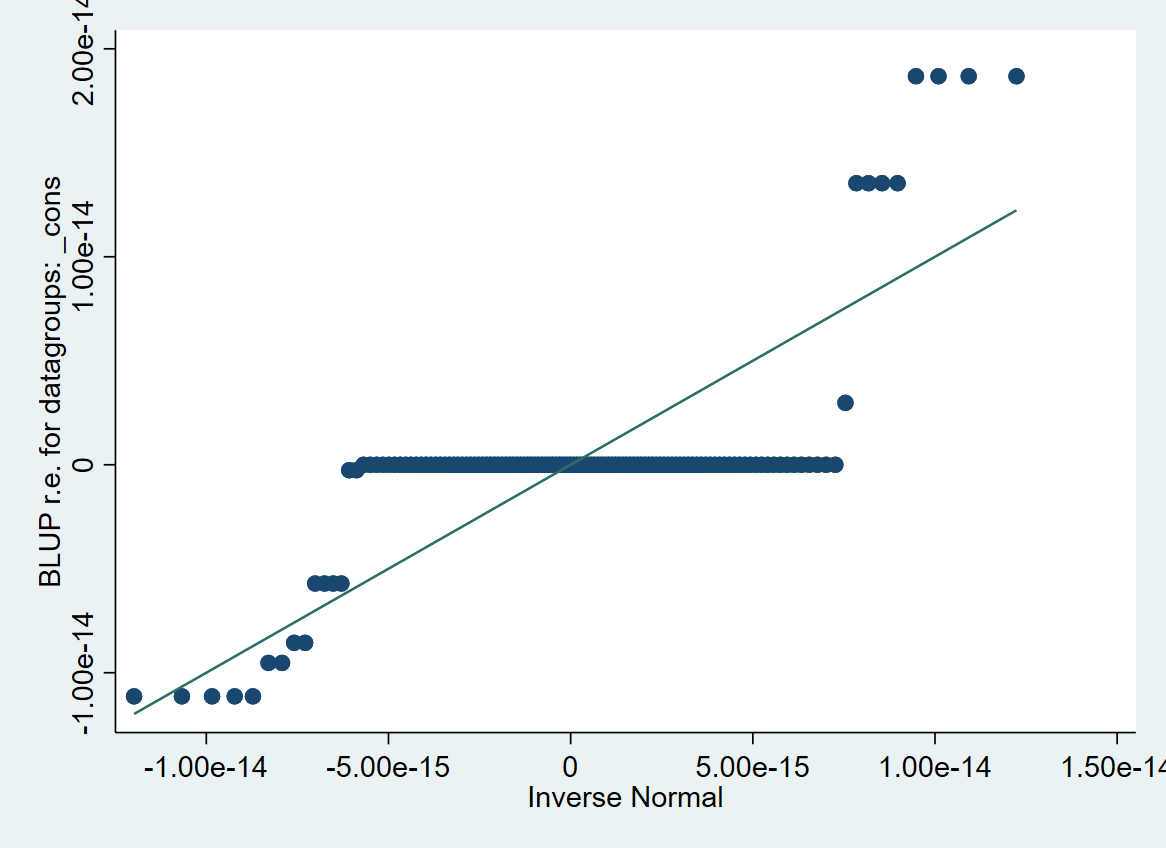


Figure 3: QQ plot of random effects
